# Case Report: Severe *Pneumocystis jirovecii* pneumonia following zuberitamab treatment in autoimmune hemolytic anemia

**DOI:** 10.3389/fimmu.2025.1546571

**Published:** 2025-03-11

**Authors:** Qian Yang, Peng Ding, Yu-xiang Liu, Kai-chen Zhang, Pei-yang Gao

**Affiliations:** ^1^ Chengdu University of Traditional Chinese Medicine, Chengdu, China; ^2^ Department of Critical Care Medicine, Hospital of Chengdu University of Traditional Chinese Medicine, Chengdu, China

**Keywords:** autoimmune hemolytic anemia, autoimmune diseases, zuberitamab, B-cell depletion, severe *Pneumocystis jirovecii* pneumonia, case report

## Abstract

The pathogenesis of autoimmune hemolytic anemia (AIHA) remains incompletely understood, typically associated with immune dysfunction and the production of autoantibodies. Zuberitamab, a novel anti-CD20 monoclonal antibody, represents an important therapeutic strategy for managing autoimmune diseases. Here, we present the first case of a patient diagnosed with AIHA who developed severe immunosuppression, lymphopenia, and B-cell depletion following zuberitamab treatment, ultimately resulting in severe *Pneumocystis jirovecii* pneumonia(PJP). This case highlights the complexities of B-cell-targeted immunotherapy and underscores the necessity of close monitoring of immune status in patients receiving zuberitamab or other targeted immunotherapies to mitigate the risk of severe immune-related adverse events.

## 1 Introduction

AIHA is a condition involving the production of antibodies against one’s own red blood cells (1–3). Autoimmune diseases are characterized by the recognition of self‐antigens by the immune system, which leads to inflammation and tissue damage. B cells are directly and indirectly involved in the pathophysiology of autoimmunity through antigen presentation to T cells and the production of proinflammatory cytokines and/or autoantibodies. Consequently, B lineage cells have been identified as therapeutic targets in autoimmune diseases (4). Anti-CD20 antibody therapy specifically targets the CD20 antigen on B cell surfaces and has been demonstrated as an effective treatment for various autoimmune diseases (5–7). Zuberitamab, a novel humanized chimeric anti-CD20 monoclonal antibody, selectively binds to CD20 antigens on B-cell surfaces, inducing B-cell depletion through antibody-dependent cellular cytotoxicity (ADCC) and complement-mediated cytotoxicity (8). While zuberitamab has shown promise in managing autoimmune diseases, its immunosuppressive effects may predispose patients to opportunistic infections.

This report presents a case of severe PJP following zuberitamab treatment in a patient with AIHA, aiming to provide practical insights and reference for evaluating the safety profile of this therapeutic approach.

## 2 Case presentation

A 65-year-old female was admitted to the hospital on May 20, 2025, due to “fatigue and dizziness for 3 days”. Laboratory tests revealed hemoglobin levels of 65 g/L (reference range: 115-150), reticulocyte percentage of 16.11% (reference range: 0.5-1.5), total bilirubin of 38.8 μmol/L (reference range: 0-21), conjugated bilirubin of 12 μmol/L (reference range: 0-8), unconjugated bilirubin of 26.8 μmol/L (reference range: 0-17), and lactate dehydrogenase (LDH) of 428 U/L (reference range: 120-250). A direct antiglobulin test (DAT) was positive, confirming the diagnosis of AIHA on May 23, 2024. Initial glucocorticoid theraphy showed limited efficacy. Subsequently, she received zuberitamab(600 mg) on July 4, July 10, July 18, and July 25, 2024, at an external hospital. The patient had no significant medical history and was previously in good health.

**Figure 1:**
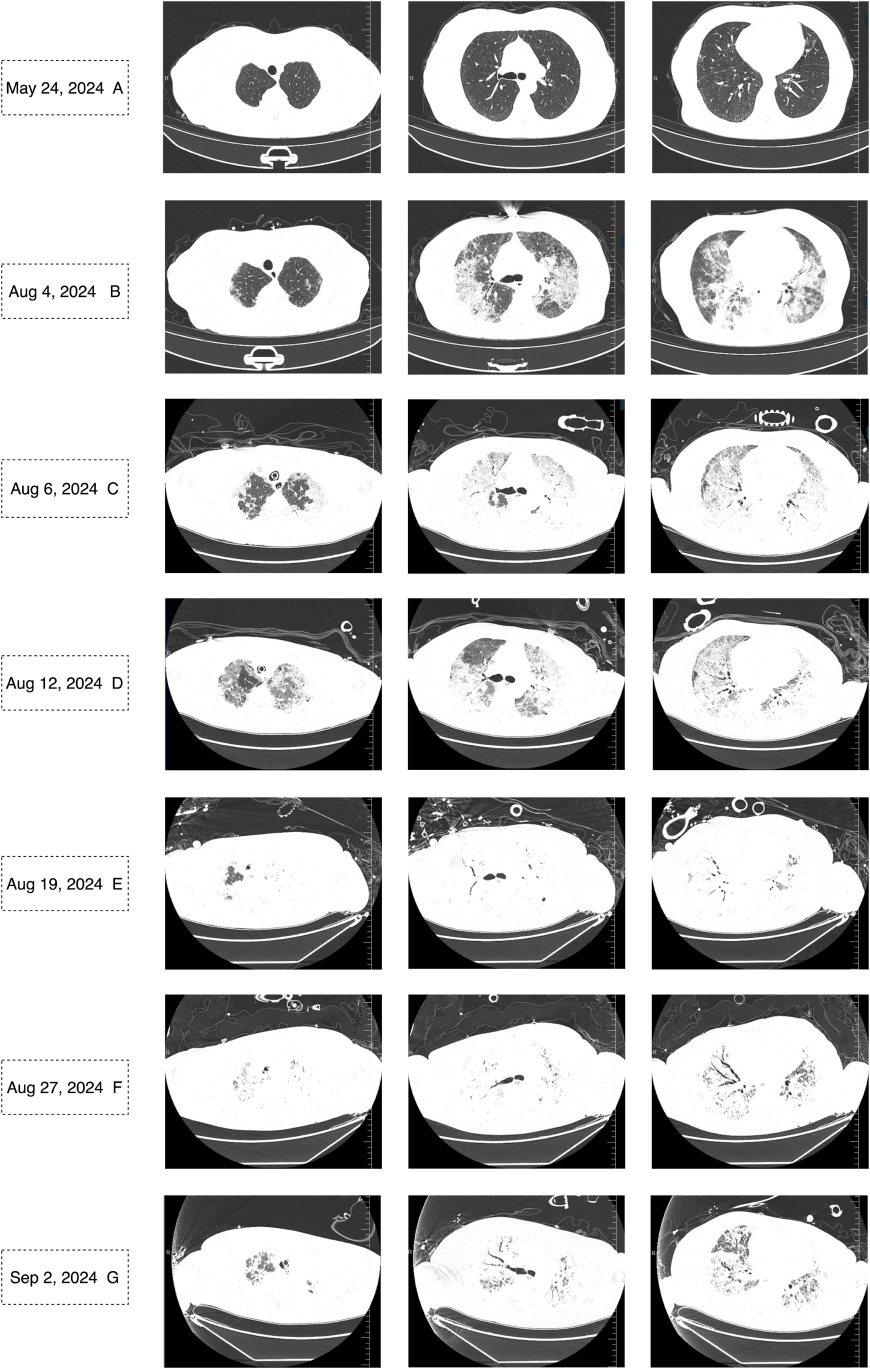
Chest CT on May 24, 2024, showed no signs of infection **(A)**. Chest CT scans from August 4 to September 2, 2024, revealed a gradually expanding infectious focus in the lungs, progressively increasing lung parenchymal density, and bronchiectasis with traction signs **(B-G)**.

**Figure 2:**
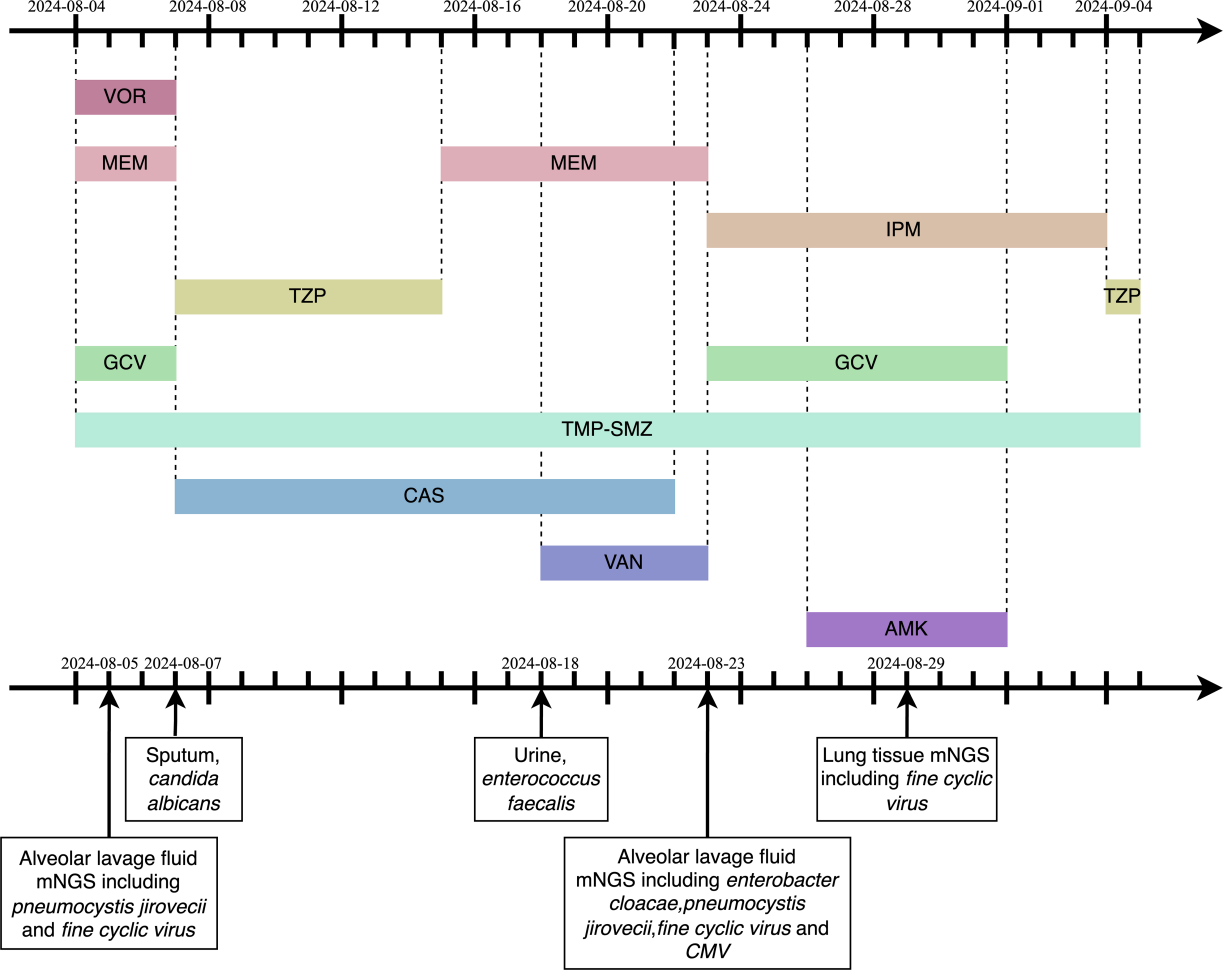
The timeline of antimicrobial medication. VOR,Voriconazole;MEM,Meropenem;IPM,Imipenem;TZP,Piperacillin/Tazobactam;GCV,Ganciclovir;mNGS,metagenomic next-generation sequencing;TMP-SMZ,trimethoprim-sulfamethoxazole;CAS,Caspofungin;VAN,Vancomycin;AMK,Amikacin; CMV, *Cytomegalovirus*.

**Figure 3:**
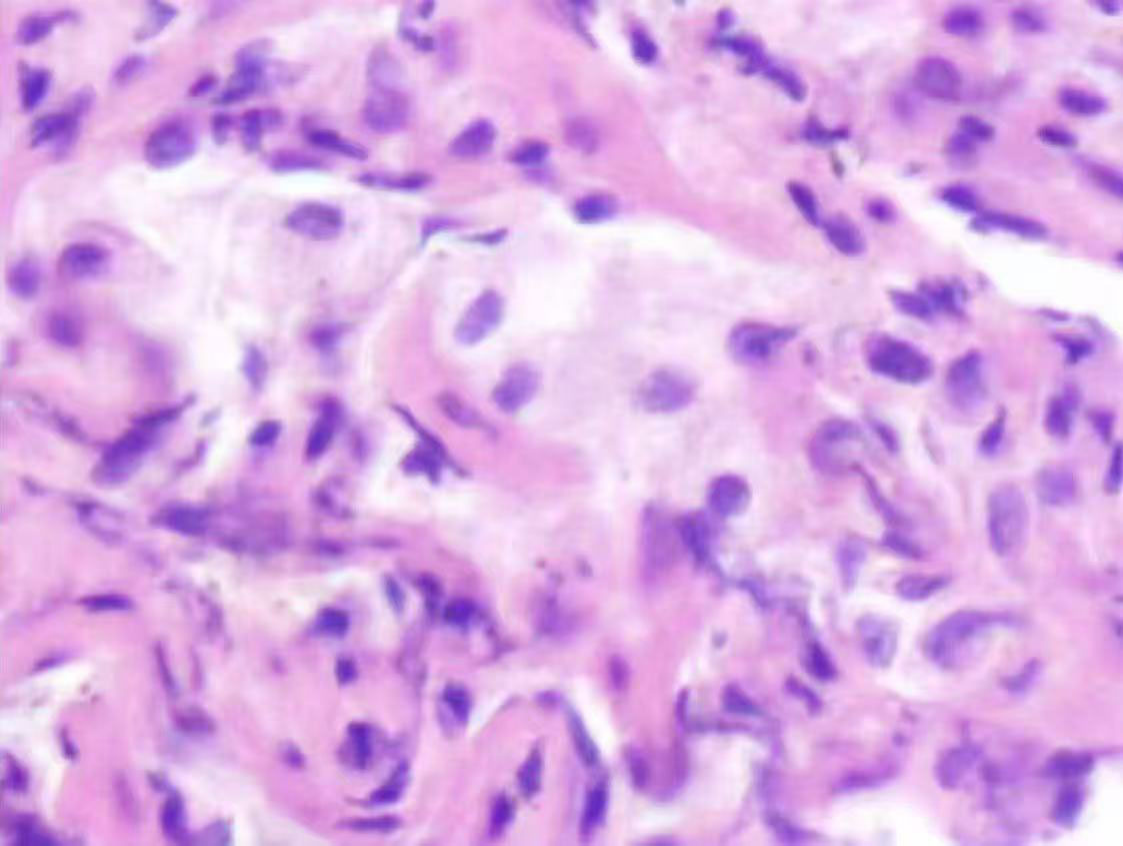
Pathological examination revealed lung parenchyma with alveolar collapse, widened alveolar septa accompanied by scattered lymphoplasmacytic infiltration, fibrous tissue hyperplasia, and focal foam-like cellular deposits within alveolar spaces.

On August 4, 2024, at 10:26 AM, the female was hospitalized with a 4-day history of fever. At 7:05 PM the same day, the female developed respiratory distress and dyspnea after using the restroom, with oxygen saturation dropping to 77% under mask oxygen therapy. The patient was subsequently transferred to the intensive care unit (ICU) for further monitoring. Arterial blood gas analysis revealed pH 7.35 (reference range: 7.35-7.45), partial pressure of oxygen (PO_2_) 76.24 mmHg (reference range: 83-108), partial pressure of carbon dioxide (PCO_2_) 47.3 mmHg (reference range: 32-48), and oxygen index (OI) 108.92 mmHg(reference range: 400-500). Immediate endotracheal intubation and mechanical ventilation were initiated. Compared to the chest Computed Tomography (CT) on May 24 (**Figure 1A**), the chest CT on August 4 showed multiple infectious lesions in both lungs (**Figure 1B**). Given the patient’s history of oral glucocorticoid therapy and immunosuppressed state, and the potential for B-cell depletion induced by zuberitamab (9), the risk of opportunistic pathogen infection was significantly elevated. Broad-spectrum antimicrobial therapy was commenced, including meropenem, sulfamethoxazole, voriconazole, and ganciclovir. On August 6, respiratory distress progressively worsened, with the oxygen index declining to 34.24 mmHg. Repeated Chest CT revealed an expanded range of pulmonary infectious lesions and increased consolidation areas (**Figure 1C**). Emergency veno-venous extracorporeal membrane oxygenation (VV-ECMO) support was implemented. On August 7, metagenomic next-generation sequencing (mNGS) of bronchoalveolar lavage fluid identified *Pneumocystis jirovecii* (sequence count: 30,899, relative abundance: 98.88%). Subsequent antifungal treatment with caspofungin was initiated, with the antimicrobial treatment timeline illustrated in **Figure 2**. On August 18, urine culture revealed *Enterococcus faecalis*, thus combination therapy with vancomycin was initiated for infection management. On August 24, mNGS of bronchoalveolar lavage fluid detected *Enterobacter cloacae complex* (sequence count: 791),*Pneumocystis jirovecii* (sequence count: 5), *fine cyclic virus*(sequence count: 2599) and *Cytomegalovirus* (sequence count: 918). Consequently, vancomycin and caspofungin were discontinued, and combination therapy with imipenem and amikacin was started for infection control. The patient exhibited persistent immunosuppression, abnormal cellular immune markers, B-cell depletion, and low helper/inducer T lymphocyte counts. Immunoenhancement therapies, including intravenous immunoglobulin and thymosin, were administered sequentially, but with unsatisfactory outcomes (**Tables 1**, **2**). Follow-up chest CT scans on August 12 and August 19 revealed an increased number of lesions in both lungs and progressively increased pulmonary parenchymal density (**Figures 1D, E**). On August 27, under deep analgesia and sedation, the patient received high-flow ECMO and invasive mechanical ventilation. The patient’s vital signs were relatively stable, but the tidal volume was low, and pulmonary imaging did not show significant improvement. Lung parenchymal density demonstrated an increasing trend, with bronchiectasis and notable traction signs observed (**Figure 1F**). Comprehensively considering the potential inflammatory proliferative phase of the patient’s disease, a lung tissue pathological diagnosis was performed to understand the disease progression and potential long-term treatment outcomes. The mNGS of the left upper lung tissue revealed a potential circovirus infection, with no concurrent fungal or bacterial pathogens identified. Pathological examination on August 30 revealed lung parenchyma with alveolar collapse, widened alveolar septa accompanied by scattered lymphoplasmacytic infiltration, fibrous tissue hyperplasia, and focal foam-like cellular deposits within alveolar spaces (**Figure 3**). Considering the possibility of organizing pneumonia, high-dose corticosteroid pulse therapy was initiated. Repeated Chest CT on September 2 demonstrated diffuse pulmonary consolidation, honeycomb-like changes, and prominent bronchial signs (**Figure 1G**).

**Table 1:** Changes in B-cell and helper/inducer T lymphocyte counts.

Marker	2024-08-06	2024-08-13	2024-08-20	2024-08-26	2024-09-02	Reference range
B-lymphocyte count (cells/uL)	1	0	0	1	1	80-616
Helper/inducer T-lymphocyte count (cells/uL)	52	40	39	125	70	404-1612

**Table 2:** Changes in lymphocyte count and lymphocyte percentage.

Marker	2024-05-20	2024-06-04	2024-08-04	2024-08-06	2024-09-05	Reference range
Lymphocytes percentage (%)	26.9	19.1	4.9	6.8	6.2	20-50
Lymphocyte count (10^9/L)	2.5	1.93	0.28	0.26	0.17	1.1-3.2

Despite aggressive comprehensive treatment, the patient continued to require VV-ECMO, with no lung function recovery and an extremely poor long-term prognosis. On September 6 at 09:27 AM, the patient’s family decided to withdraw treatment, and clinical death was pronounced at 10:22 AM. The cause of death was acute respiratory distress syndrome (ARDS).

## 3 Discussion

This case reports a patient with autoimmune hemolytic anemia who developed B lymphocyte depletion and severe immunodeficiency following treatment with zuberitamab, ultimately resulting in severe PJP. Despite the medical team’s aggressive treatment measures, the patient unfortunately succumbed to the condition. This case reveals the potential risks of severe immune-related adverse events (irAEs) associated with zuberitamab theraphy in patients with AIHA.

AIHA is a complex autoimmune disorder characterized by immune dysfunction, where autoreactive antibodies target erythrocytes, resulting in their progressive destruction (10–12). Anti-CD20 monoclonal antibodies have emerged as an effective therapeutic strategy for AIHA (2). Zuberitamab, a humanized chimeric IgG1 anti-CD20 monoclonal antibody, effectively eliminates B cells through ADCC and complement-mediated cytotoxicity (8). Relevant research demonstrated that a single 100 mg dose of zuberitamab can rapidly and completely eliminate B cells within one week, with B-cell depletion sustained for at least 24 weeks (13). While this mechanism is highly effective in suppressing aberrant immune responses, it can also severely compromise immune defenses, leaving patients vulnerable to opportunistic infections such as PJP. Notably, PJP in non-HIV patients is often more severe, with higher mortality rates (14, 15). Emerging evidence has underscored the critical role of B cells in clearing Pneumocystis infections. Beyond their role as antibody producers, B cells are vital for antigen presentation, T-cell proliferation, and overall immune defense, constituting a critical component of the immune system (16–21).

Current guidelines do not explicitly mandate PJP prophylaxis for anti-CD20 antibody monotherapy. However, initiating preventive treatment becomes necessary when high-dose corticosteroids (e.g., prednisone dose exceeding 20 mg/day for more than one month), absolute lymphocyte reduction, or other immunosuppressive conditions (22). A prospective study revealed that the majority of PJP patients admitted to intensive care units had not received prophylactic antibiotics, with up to 88% lacking preventive treatment, significantly increasing their risk of infection (23). Infection remains a well-documented complication of AIHA and a leading cause of mortality, yet there are no specific guidelines for infection prevention in this patient population (24). Prophylactic anti-PJP medication, such as trimethoprim-sulfamethoxazole (TMP-SMX), may represent an effective intervention strategy (25). Antibiotic prevention for PJP represents a validated intervention, with prevention deficiency recognized as a well-established risk factor for severe PJP. For immunocompromised patients, prophylactic antibiotic treatment should be considered to reduce the morbidity and mortality associated with PJP (26–28).

Here, this case demonstrates the potential risk of severe immunodeficiency and opportunistic infections associated with zuberitamab treatment in AIHA. While zuberitamab effectively controls the aberrant immune response in AIHA through B-cell depletion, the concomitant weakening of immune defense mechanisms can lead to fatal consequences. B cells play a pivotal role not only in antibody production and antigen presentation but also in maintaining the immune system's overall integrity and infection-fighting capacity. Moreover, there are currently no established guidelines for infection prophylaxis in AIHA patients, and the lack of specific strategies for PJP prevention may further increase the risk of infections. One limitation of this case is the lack of further characterization of the AIHA subtype, which could provide more clarity for targeted treatment strategies. We recommend conducting rigorous and comprehensive assessments of patients undergoing B cell-targeted immunotherapy. These measures are essential to enhancing therapeutic efficacy while ensuring patient safety and quality of life. However, further research and additional cases are required to validate these observations and guide clinical practice.

## Data Availability

The original contributions presented in the study are included in the article/supplementary material. Further inquiries can be directed to the corresponding author.
